# Whole-genome resequencing reveals the genetic diversity, population structure and selection signatures in Chinese indigenous Kele pigs

**DOI:** 10.3389/fvets.2025.1655561

**Published:** 2025-10-29

**Authors:** Yixuan Zhu, Xiaoyi Wang, Ligang Lu, Yongli Yang, Qiang Chen, Chengliang Xu, Jinhua Lai, Lixing Wang, Shuyan Wang, Mingli Li, Shaoxiong Lu

**Affiliations:** 1 Faculty of Animal Science and Technology, Yunnan Agricultural University, Kunming, China; 2 Bijie Academy of Agricultural Sciences, Bijie, China; 3 Yunnan Provincial Livestock Station, Kunming, China

**Keywords:** Kele pig, genetic diversity, population structure, selection signature, whole-genome resequencing

## Abstract

**Introduction:**

Kele pig (KLP) is a valuable Chinese indigenous pig breed, renowned for its strong adaptability, high intramuscular fat content, and excellent meat quality. However, the genomic characteristics of KLPs are still unknown. This study aims to investigate the genetic diversity, population structure, and trait-related selection signatures of KLPs based on whole-genome resequencing.

**Methods:**

The genomes of 30 KLPs were resequenced and analyzed alongside genomic data from 90 pigs of three commercial breeds, comprising 30 Duroc (DUPs), 30 Landrace (LRPs), and 30 Yorkshire pigs (YRPs). To evaluate their genetic diversity, we calculated the expected heterozygosity, observed heterozygosity, polymorphic marker ratio, minor allele frequency, nucleotide diversity (π), runs of homozygosity (ROH), and inbreeding coefficient (F_ROH_). Meanwhile, a neighbor-joining tree, principal component analysis, ADMIXTURE analysis, linkage disequilibrium (LD) analysis, genetic distance and relationship matrices were constructed to analyze the population structure. In addition, selection signatures between KLPs and DUPs, LRPs, and YRPs were detected using fixation index (Fst) and π ratio methods.

**Results and Discussion:**

A total of 66,204,339 autosomal single nucleotide polymorphisms (SNPs) were detected in the 120 pigs, and 21,738,497 SNPs were retained for further analysis after filtering. The results showed that KLPs had higher genetic diversity, along with the smallest F_ROH_ value compared to DUPs, LRPs, and YRPs. Moreover, KLPs displayed a relatively unique genetic structure with a higher LD decay, and the majority of individuals within the KLPs exhibited distant genetic distances and relationships. Totals of 688 selected regions were identified, including 723 published QTLs. Within the selected regions, 192 candidate genes were annotated, and seven genes were found to be functionally involved in coat color (*KIT*), immune response (*JAK2* and *SOCS1*), heart development (*NTRK3* and *SRF*), muscle growth and development (*VDR*), and fat deposition (*KDR*). These findings will provide valuable insights for the future conservation, breeding, and utilization of KLPs.

## 1 Introduction

China possesses one of the world’s richest diversities of indigenous pig breeds, resulting from long-term domestication and selection under diverse ecological-geographic conditions and traditional ethnic cultures. These diverse breeds provide valuable germplasm resources for the sustainable development of pig industry. Compared to commercial pig breeds, Chinese indigenous pig breeds generally exhibit advantages such as strong adaptability, resistance, and superior meat quality, but also have some drawbacks, including slower growth rates and higher carcass fat content (1). In pursuit of higher production efficiency, extensive crossbreeding has been conducted between introduced commercial pig breeds and Chinese indigenous breeds in recent decades, posing severe threats to indigenous breeds and resulting in a sharp decline in both breed number and population sizes (2). Therefore, it is particularly urgent and necessary to strengthen the protection, breeding, and utilization of the genetic resources of Chinese indigenous pig breeds.

In recent years, the development of DNA sequencing technology has facilitated the efficient detection of genomic variations, providing a more accurate powerful tool for population genetic studies in pigs. Some studies have investigated the genetic diversity, population structure, and selection signatures using genomic variation in pigs (3–5). These results have further revealed the evolutionary history and population structure, and effectively identified many selected regions and candidate genes associated with important economic traits in different pig breeds. This is of great significance for promoting the scientific conservation and optimized breeding of indigenous pig breeds.

Kele pig (KLP) is a typical indigenous pig breed in southwest China, primarily distributed in the high-altitude mountainous regions of northwestern Guizhou Province, at elevations ranging from 1,700 to 2,400 meters. Due to the long-term local domestication, rearing, and selection, KLPs have developed several distinctive characteristics, including unique physical characteristics, strong adaptability and resistance, as well as high intramuscular fat (IMF) content and superior meat quality (6, 7). However, KLPs also exhibit some notable limitations, such as slower growth rates and lower lean meat percentages (8). Similar to other indigenous pig breeds, the population size of KLPs has also been declining in recent years due to extensive crossbreeding practices. Consequently, it is particularly imperative to enhance the conservation and utilization of KLPs based on the understanding of their population genetic characteristics. But to date, research on KLPs remains scarce. The majority of available studies have primarily focused on phenotypic traits and candidate gene analyses, with only a few investigations employing DNA microarray genotyping to examine population characteristics (4). Notably, comprehensive assessments of their genetic diversity, population structure, and selection signatures using genome-wide resequencing approaches are still lacking.

Comparing the genomes of Chinese indigenous pig breeds with those of commercial pig breeds can not only provides insights into the genetic differences caused by their distinct selection histories but also reveals potential genetic introgression resulting from the long-term introduction of commercial breeds. In this study, we performed whole-genome resequencing of KLPs and compared their genomic data with those of three commercial pig breeds: Duroc (DUP), Landrace (LRP), and Yorkshire (YRP). Using genome-wide single nucleotide polymorphisms (SNPs), a comprehensive analysis was conducted to investigate the genetic diversity and population structure of KLPs. Additionally, the fixation index (Fst) and nucleotide diversity (π) ratio methods were employed to identify putative selection signatures, including specific genomic regions and candidate genes under selection in KLPs. The present study aims to further enhance our understanding of the genomic characteristics of KLPs, thereby providing valuable insights for future optimization of their conservation and breeding.

## 2 Materials and methods

### 2.1 Sample collection, DNA extraction, and sequencing

A total of 30 unrelated KLPs were selected and their ear tissue samples were collected. Genomic DNA was extracted from the ear tissues using the TIANamp Genomic DNA Kit (Tiangen, China). The quality of the genomic DNA was evaluated using the Agilent 5400 analysis system (Agilent, United States) and 1% agarose gel electrophoresis. DNA libraries (paired-end, 2 × 150 bp) were then constructed for all samples and sequenced using the DNBSEQ-T7 platform (Novogene Bioinformatics Technology Co., Ltd., Beijing, China). Genomic data from 90 pigs of three commercial breeds (30 DUPs, 30 LRPs, and 30 YRPs) were downloaded from the NCBI SRA database.^1^ The accession numbers are listed in Supplementary Table S1. In total, genomic data from 120 pigs of four breeds were used in this study.

### 2.2 SNP detection and annotation

Raw resequencing reads were initially filtered using fastp v0.23.2 (9) to obtain clean reads. Clean reads were then mapped to the reference genome (*Sus scrofa* 11.1) using BWA v0.7.17 (10), and sorted binary bam files were obtained using SAMtools v1.6 (11). Subsequently, Picard tools were used to filter out possible duplicate reads (REMOVE_DUPLICATES = true). SNP detection was performed using the Genome Analysis Toolkit (GATK v4.4.0) (12). Raw SNPs were detected using the “HaplotypeCaller,” “GenotypeGVCFs,” and “SelectVariants” modules of GATK and then filtered using the parameters “QD < 2.0, MQ < 40.0, FS > 60.0, SOR > 3.0, MQRankSum < −12.5 and ReadPosRankSum < −8.0.” SNPs were annotated based on the *Sus scrofa* 11.1 genome (GCF_000003025.6) using ANNOVAR v2.0 (13) with the parameters (−annotate_variation.pl. -dbtype refGene). Finally, VCFtools v0.1.16 (14) was used for further filtering with the following parameters: “--min-alleles 2 --max-alleles 2 --maf 0.05 --max-missing 0.1,” and the filtered SNPs were used for subsequent analysis.

### 2.3 Genetic diversity analysis

The expected heterozygosity (H_E_), observed heterozygosity (H_O_), polymorphic marker ratio (P_N_), and minor allele frequency (MAF) were calculated using PLINK v1.9 (15). The π value was calculated using VCFtools v0.1.16 (14). Runs of homozygosity (ROH) were calculated using PLINK v1.90 (15) with the following parameters: “--homozyg-density 50 --homozyg-gap 1,000 --homozyg-kb 500 --homozyg-snp 50 --homozyg-window-het 1 --homozyg-window-snp 50 --homozyg-window-threshold 0.05.” The ROH of each population was classified into five categories (0.5 ~ 1 Mb, 1 ~ 2 Mb, 2 ~ 3 Mb, 3 ~ 4 Mb, and > 4 Mb). Besides, the genomic inbreeding coefficient based on ROH (F_ROH_) was calculated for each population.

### 2.4 Population structure analysis

The distance matrix was calculated using VCF2Dis v1.50^2^, and a neighbor-joining (NJ) tree was constructed based on the matrix using FastME 2.0^3^ and visualized using the ggtree package (16). Principal component analysis (PCA) was performed using PLINK v1.90 (14) with the parameter (--pca 10), and the first two dimensions were used to distinguish population structure. Population structure was analyzed using ADMIXTURE v1.3.0 (17), and ancestral population number (K) was set from 1 to 8. Visualization of the ancestry composition was performed using the R package of Pophelper (18). Linkage disequilibrium (LD) decay with physical distance between SNPs was calculated and visualized using PopLDdecay v3.42 (19) with the default parameters.

### 2.5 Genetic distance and relationship analysis

An identity by state (IBS) matrix was constructed using PLINK v1.9 (15) to analyze the genetic distance between individuals within KLPs. Additionally, a genomic relationship (G) matrix was constructed using GCTA v1.94 (20) to analyze the genetic relationship between individuals within KLPs. To improve the intuitiveness of the numerical distribution, the elements of the G matrix were normalized to the range from −1 to 1 and visualized using the R package of pheatmap.

### 2.6 Selection signature analysis

The Fst and π ratio methods were used to detect the selection signatures in KLPs compared to DUPs, LRPs, and YRPs. The four pig populations were divided into three comparisons: KLPs vs. DUPs, KLPs vs. LRPs, and KLPs vs. YRPs. Fst and π ratio values were calculated for each comparison using 100 kb sliding windows with 10 kb steps in VCFtools v0.1.16 (14). The overlapping windows in the top 5% threshold of the Fst and π ratio values for each comparison were considered as the selected regions. Additionally, to identify the overlap between the selected regions and published quantitative trait loci (QTLs), a total of 55,688 QTLs from 407 different traits were downloaded from the Pig QTL database (https://www.animalgenome.org/cgi-bin/QTLdb/SS/index, Release 54, 25 Aug 2024) for comparison. Moreover, candidate genes in these selected regions were annotated using the UCSC database.^4^

### 2.7 Functional enrichment analysis

To further explore the biological functions of the candidate genes, GO and KEGG enrichment analyses were performed using clusterProfiler (21) and Pathview (22) packages. The GO terms included three categories: biological process (BP), cellular component (CC), and molecular function (MF). Only those terms and pathways with *p* value < 0.05 were considered significant.

## 3 Results

### 3.1 Summary statistics of genomic data and SNPs

**Figure 1:**
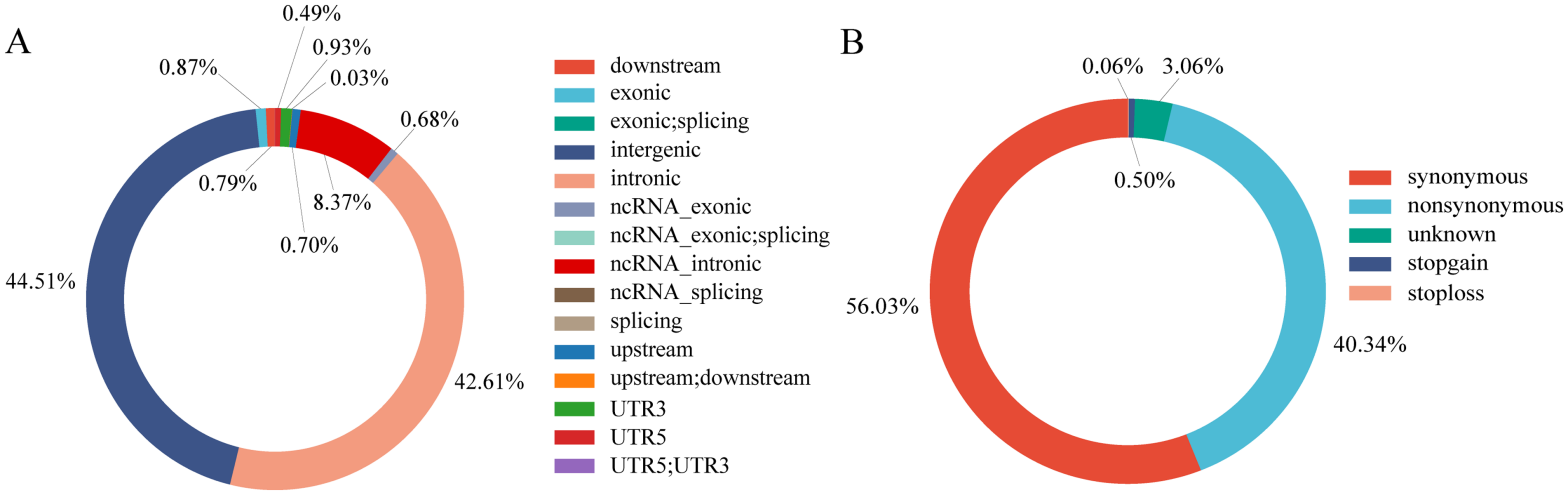
Information of the annotated SNPs. **(A)** Distribution of genomic regions of the annotated SNPs. Different colors represent different regions. **(B)** Types and proportions of the annotated SNPs within the coding region. Different colors represent different types of SNP.

A total of 1073.30 Gb of raw data was obtained for the 30 KLPs genome, and the average depth and mapping rate were 11.45 × and 98.29%, respectively. The genomic data from the 120 pigs generated more than 50 billion raw reads, of which more than 48 billion were clean reads (Supplementary Table S1). Totals of 66,204,339 autosomal SNPs were detected in the 120 pigs, and the density distribution of SNPs across the chromosomes was shown in Supplementary Figure S1. The majority of SNPs were located in intergenic (44.51%) and intronic (42.61%) regions, with a small percentage located in exonic regions (0.87%) (Figure 1A). Most of the SNPs were synonymous (56.03%) and nonsynonymous (40.34%) mutations (Figure 1B). After filtering, 21,738,497 SNPs were retained for analysis of the genetic diversity, population structure, and selection signatures.

### 3.2 Genetic diversity of KLPs

**Figure 2:**
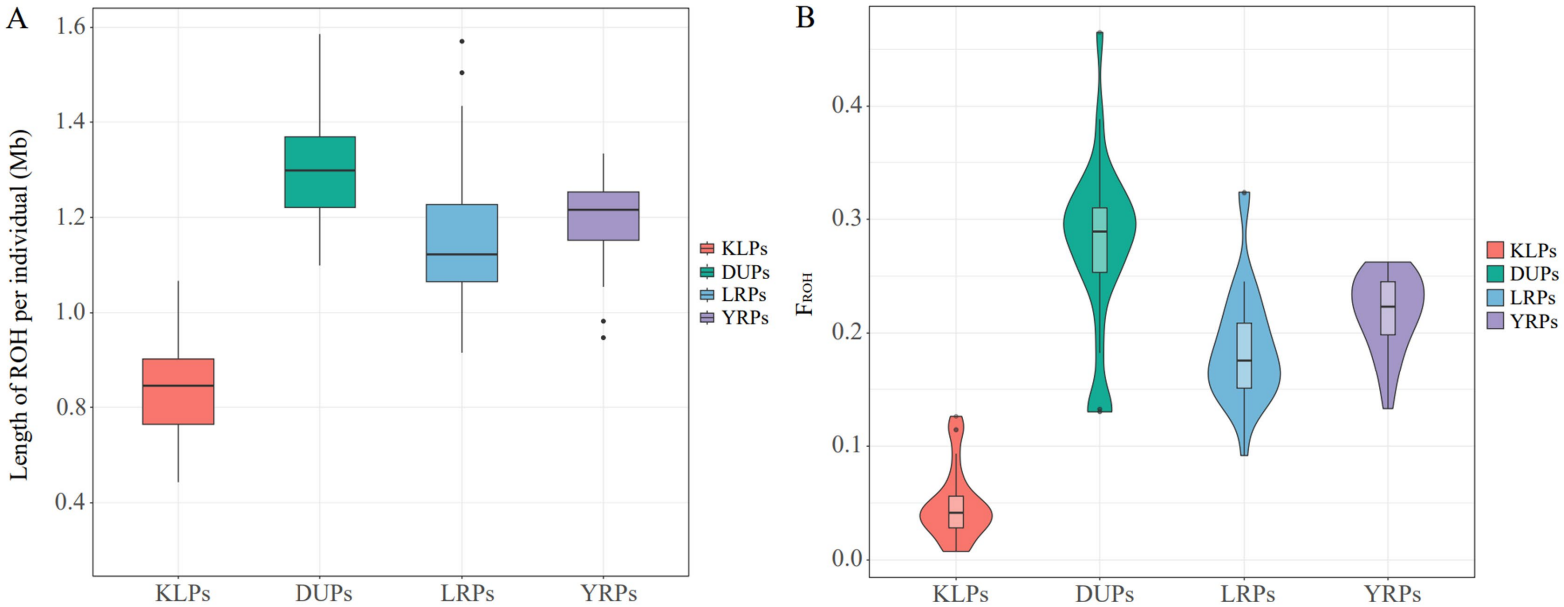
Distribution of the average lengths of ROH and values of F_ROH_ in the four pig populations. **(A)** Average lengths of ROH. **(B)** F_ROH_.

In general, the H_E_ (0.3189), H_O_ (0.3046), P_N_ (0.9425), MAF (0.2381), and π (0.2696) of KLPs were higher than those of DUPs, LRPs, and YRPs (Table 1). The H_O_ was lower than H_E_ in the four pig populations. Besides, a total of 40,321 ROHs were identified in 119 pigs, and no one was detected in one individual (K30). KLPs had the minimum number of ROH among the four populations, and most of the ROH were mainly concentrated in 0.5 ~ 1 Mb, followed by 1 ~ 2 Mb (Table 2). Besides, compared with the three commercial pig breeds, KLPs had the shortest length of ROH per individual and the smallest F_ROH_ value (Figures 2A,B). The F_ROH_ in KLPs was ranged from 0.0075 to 0.1262, and the average F_ROH_ was 0.0479.

**Table 1:** The genetic variation of the four pig populations.

Population	N	H_E_	H_O_	P_N_	MAF	π
KLPs	30	0.3189	0.3046	0.9425	0.2381	0.3243
DUPs	30	0.2008	0.1859	0.6829	0.1461	0.2042
LRPs	30	0.2260	0.1967	0.8022	0.1623	0.2298
YRPs	30	0.2344	0.2247	0.7670	0.1718	0.2384

N, number of samples; H_E_, expected heterozygosity; H_O_, observed heterozygosity; P_N_, polymorphic marker ratio; MAF, minor allele frequency; π, nucleotide diversity; KLPs, Kele pigs; DUPs, Duroc; LRPs, Landrace; YRPs, Yorkshire.

**Table 2:** The distribution of ROH in the four pig populations.

Population	N	Number of different length of ROH					
		0.5 ~ 1 Mb	1 ~ 2 Mb	2 ~ 3 Mb	3 ~ 4 Mb	>4 Mb	Total
KLPs	29	2,317	589	83	21	12	3,022
DUPs	30	7,766	4,065	1,287	474	508	14,099
LRPs	30	6,619	2,979	764	262	247	10,871
YRPs	30	6,963	3,803	1,022	317	224	12,329

N, number of samples; KLPs, Kele pigs; DUPs, Duroc; LRPs, Landrace; YRPs, Yorkshire.

### 3.3 Population structure of KLPs

**Figure 3:**
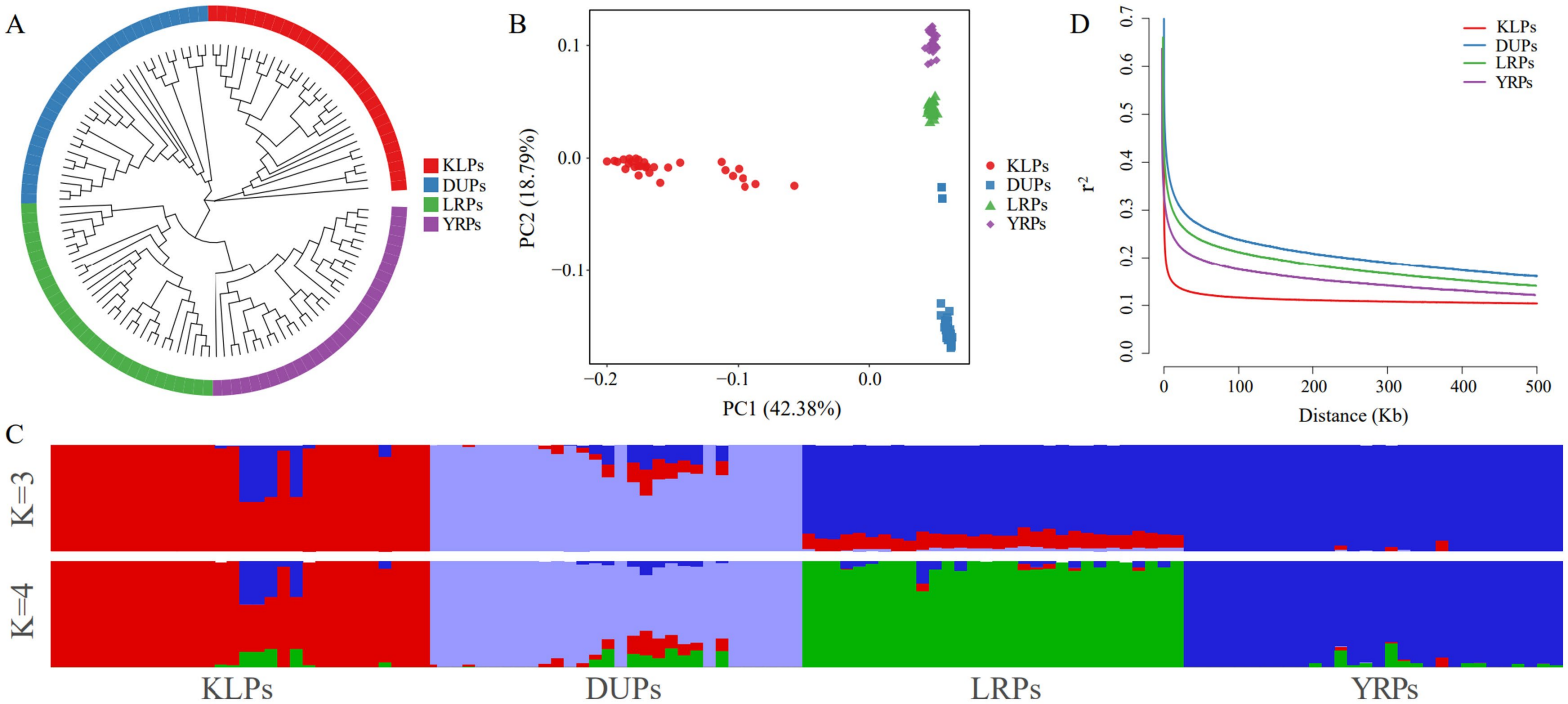
Population structure of KLPs and the three commercial pig breeds. **(A)** Neighbor-joining tree constructed from SNP data among the four pig populations. **(B)** Principle component analysis for the first two PCs of the 120 pigs. **(C)** ADMIXTURE analysis with K = 2 and K = 3. **(D)** LD decay curves of the four pig populations.

The NJ tree showed that all KLP individuals formed a cluster, while the DUPs, LRPs, and YRPs formed a large clade (Figure 3A). However, there were multiple branches in the KLPs. The PCA also clearly distinguished the KLPs from DUPs, LRPs, and YRPs (Figure 3B). The first eigenvector (PC1) explained 42.38% of the total genetic variation, and clearly distinguished the KLPs from DUPs, LRPs, and YRPs. The second eigenvector (PC2) explained 18.79% of the total genetic variation, and clearly separated the DUPs, LRPs, and YRPs. In the KLPs, 80% of the individuals were tightly clustered together, while the remaining ones were relatively scattered. Based on the results of the ADMIXTURE analysis, K = 4 was found to be the minimum cross-validation error (Supplementary Figure S2). At K = 4, KLPs and the three commercial pig breeds were clearly distinguished, and KLPs were found to have a small amount of genetic components from LRPs and YRPs (Figure 3C). Additionally, KLPs showed a higher LD decay compared to DUPs, LRPs, and YRPs (Figure 3D).

### 3.4 Genetic distance and relationship among the individuals

**Figure 4:**
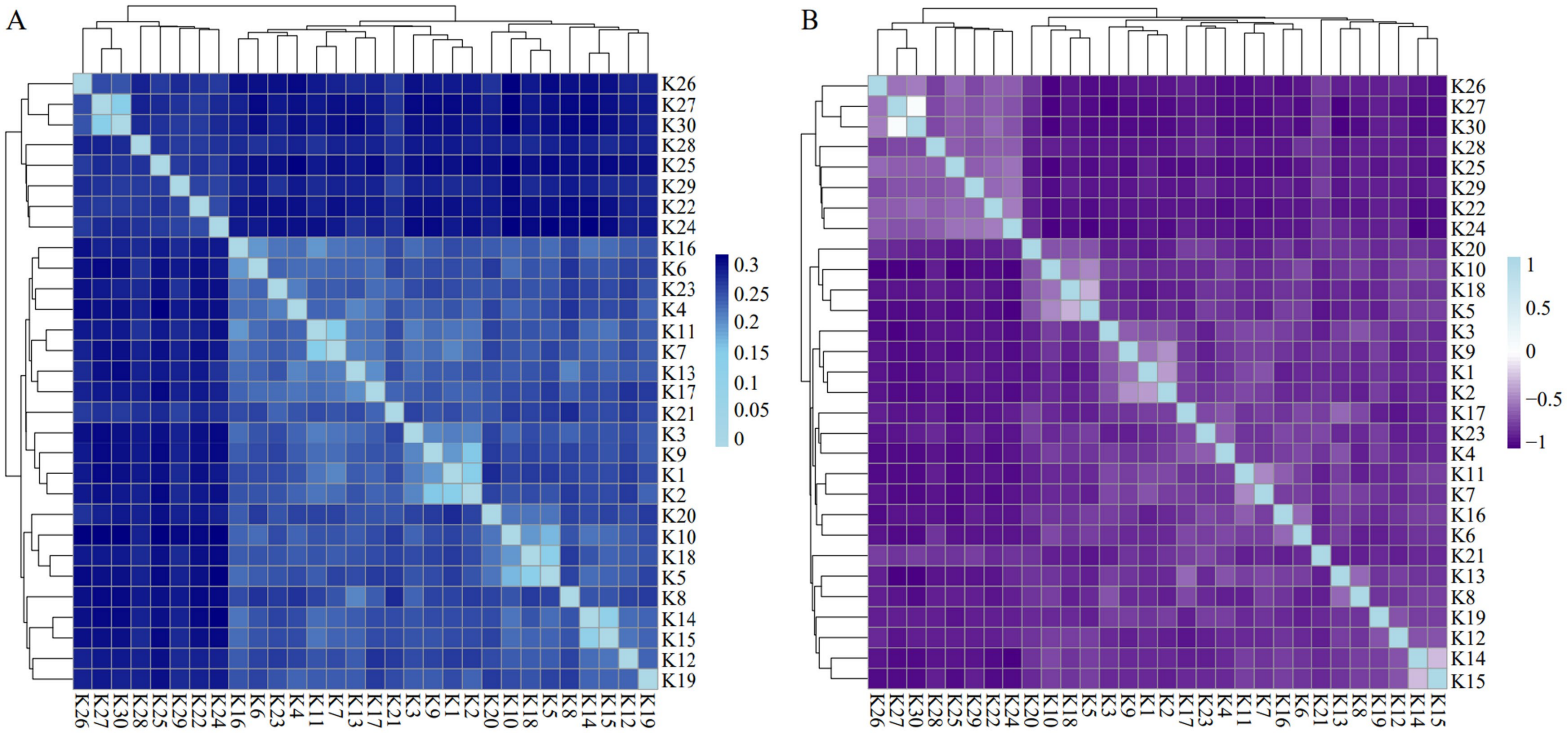
The heat map of the IBS distance and G matrices of KLPs. **(A)** The IBS distance matrix of KLPs. Each small square represents the genetic distance between the two individuals, which the color blue from light to dark indicates the genetic distance from low to high. **(B)** The G matrix of KLPs. Each small square represents the value of the genetic relationship between the two individuals, which the colors blue and purple from light to dark represent the value ranges from 0 to 1 and 0 to −1, respectively.

Among the KLPs, pairwise genetic distances between individuals ranged from 0.1188 to 0.3167, with a mean value of 0.2664. The results of the IBS distance and G matrices indicated that most of the individuals in KLPs were distant, and few individuals were close to each other (Figures 4A,B). Furthermore, all individuals were clustered in multiple branches.

### 3.5 Selection signatures detection and gene functional analysis

**Figure 5:**
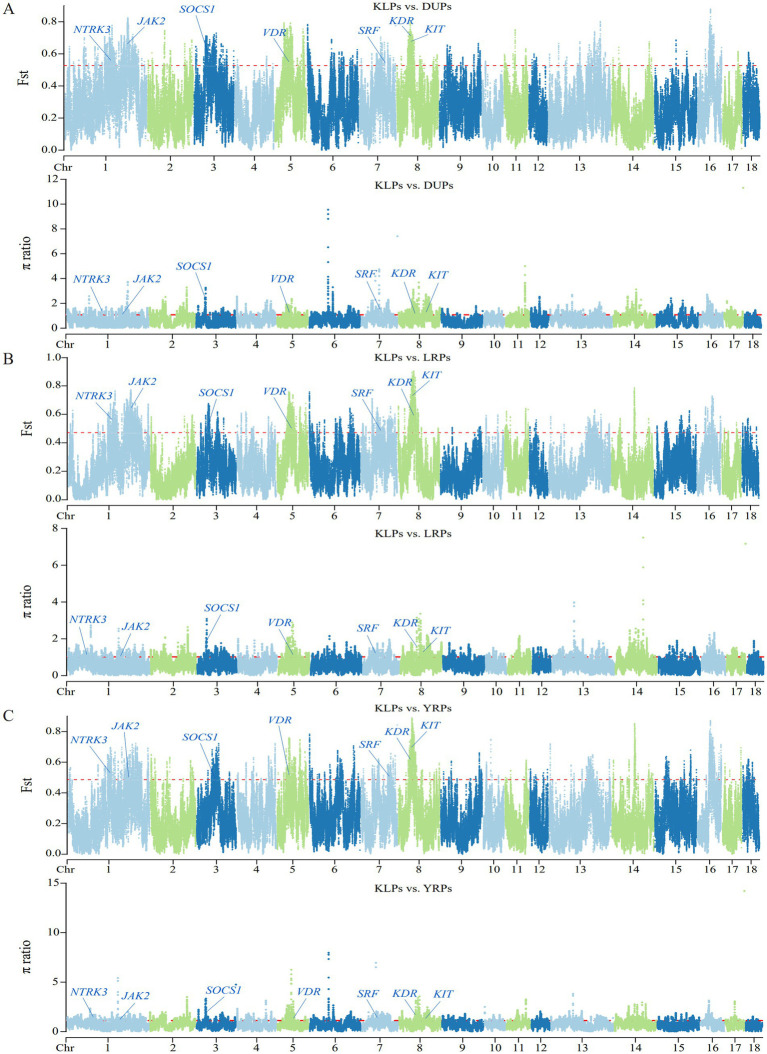
Manhattan plots of selection signatures by Fst and π ratio methods among autosomal chromosomes. The red line represents the level of 0.05. **(A)** Distribution of Fst and π ratio values in KLPs vs. DUPs comparison. **(B)** Distribution of Fst and π ratio values in KLPs vs. LRPs comparison. **(C)** Distribution of Fst and π ratio values in KLPs vs. YRPs comparison.

**Figure 6:**
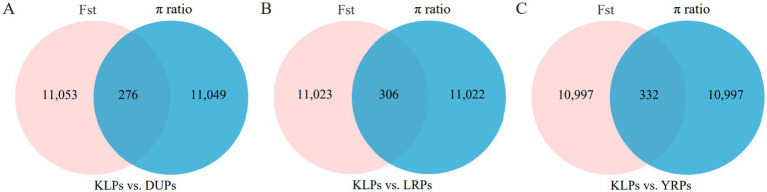
The Venn diagram of selected regions detected by the three comparisons. **(A)** Number of selected regions in KLPs vs. DUPs comparison. **(B)** Number of selected regions in KLPs vs. LRPs comparison. **(C)** Number of selected regions in KLPs vs. YRPs comparison. Each colored circle represents the number of selected regions using Fst or π ratio method.

The Manhattan plots of the distribution of Fst and π ratio values among autosomal chromosomes for the comparisons of KLPs with the three commercial pig breeds are shown in Figure 5. In the comparisons of KLPs with DUPs, LRPs, and YRPs, 276 (Fst ≥ 0.5273 and π ratio ≥ 1.0782), 306 (Fst ≥ 0.4714 and π ratio ≥ 1.0175), and 332 (Fst ≥ 0.4865 and π ratio ≥ 1.1188) windows were identified, respectively, covering 6.69 Mb, 8.41 Mb, and 10.73 Mb of the genome (Figure 6; Supplementary Tables S2–S4). Combining the three comparisons, a total of 688 selected regions were identified, covering 19.03 Mb of the genome (Supplementary Table S5). These selected regions were unevenly distributed across chromosomes (chr), with the majority of the regions were located on chr 8 and 1 (211 and 209 regions, respectively), while no regions were found on chr 9, 17, and 18. Moreover, totals of 723 published QTLs (Supplementary Table S6) were identified as being within or overlapping with the 688 selected regions. Among the 723 QTLs, 18 were associated with behavior and morphological traits (such as coping behavior and ear area), 34 with immune and health (such as basophil percentage, CD3- and CD8-negative leukocyte percentage, and melanoma susceptibility), 14 with growth (such as average daily gain and feed conversion rate), 27 with reproduction (such as litter size, piglets born alive, and age at puberty), 292 with carcass traits (such as lean cut percentage, number of ribs, and longissimus muscle area), and 338 with fat deposition and meat quality traits (such as IMF content and meat color).

**Figure 7:**
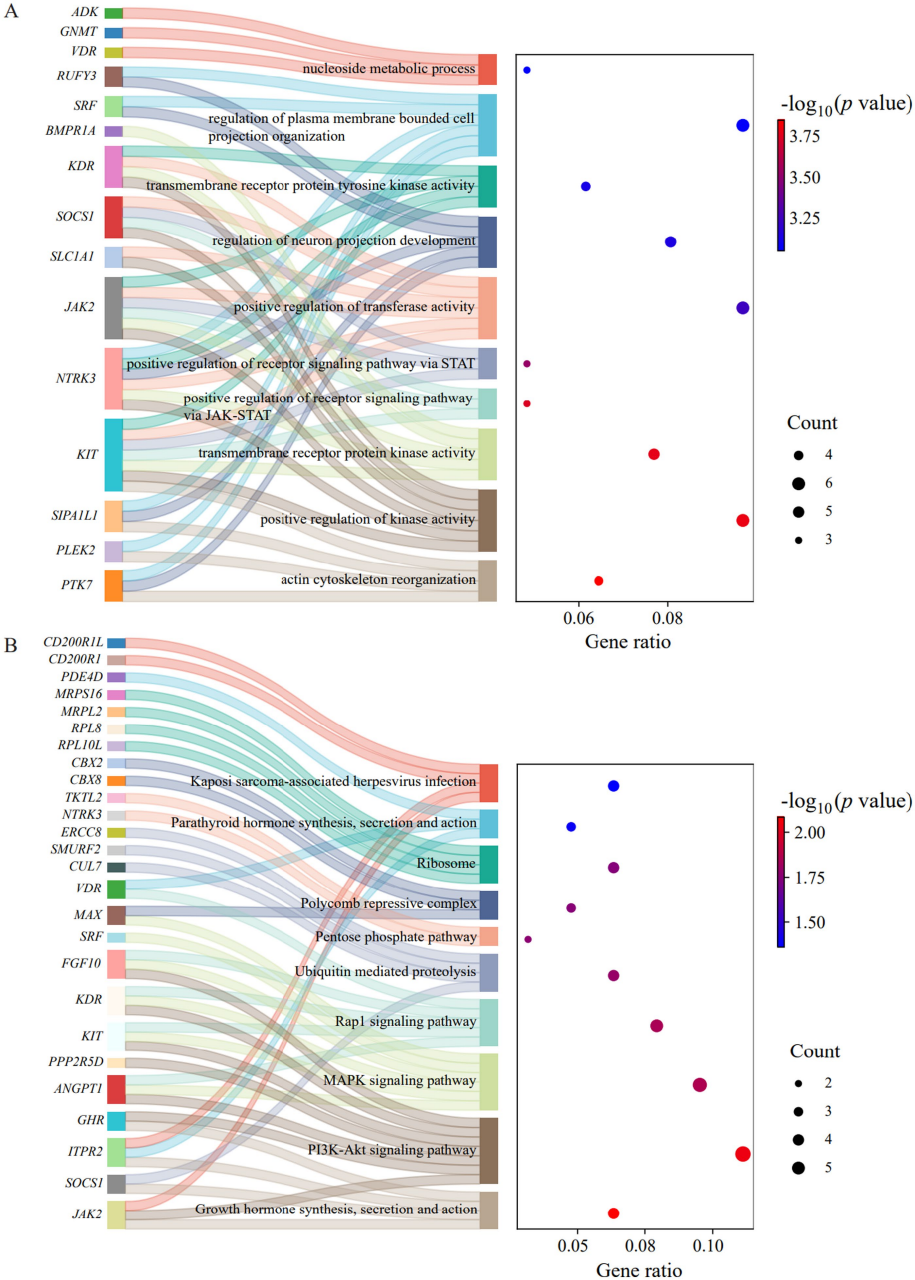
Functional enrichment analyses of the candidate genes. **(A)** The top 10 GO terms with the smallest *p* values. **(B)** The top 10 KEGG pathways with the smallest *p* values.

A total of 192 candidate genes were annotated within these selected regions (Supplementary Table S7), which covered 212 published QTLs associated with behavior and morphological, immune and health, growth, reproduction, carcass, and fat deposition and meat quality traits (Supplementary Table S8). Functional enrichment analysis of the candidate genes showed that 35 genes were significantly enriched (*p* < 0.05) in 127 BPs, 8 CCs, and 11 MFs (Supplementary Table S9). In the GO analysis, 15 genes were enriched in the top 10 GO terms with the smallest *p* values (Figure 7A), including actin cytoskeleton reorganization (GO:0031532), positive regulation of kinase activity (GO:0033674), transmembrane receptor protein kinase activity (GO:0019199), positive regulation of receptor signaling pathway via JAK–STAT (GO:0046427), positive regulation of receptor signaling pathway via STAT (GO:1904894), positive regulation of transferase activity (GO:0051347), regulation of neuron projection development (GO:0010975), transmembrane receptor protein tyrosine kinase activity (GO:0004714), regulation of plasma membrane bounded cell projection organization (GO:0120035), and nucleoside metabolic process (GO:0009116). In the KEGG analysis, 26 genes were significantly enriched in 10 pathways (*p* < 0.05) (Figure 7B; Supplementary Table S10), including growth hormone synthesis, secretion and action (ssc04935), PI3K-Akt signaling pathway (ssc04151), MAPK signaling pathway (ssc04010), Rap1 signaling pathway (ssc04015), ubiquitin mediated proteolysis (ssc04120), pentose phosphate pathway (ssc00030), polycomb repressive complex (ssc03083), ribosome (ssc03010), parathyroid hormone synthesis, secretion and action (ssc04928), and kaposi sarcoma-associated herpesvirus infection (ssc05167).

Among the significantly enriched genes, seven genes under selection were shared between the top 10 GO terms and KEGG pathways, including *KIT*, *JAK2*, *SOCS1*, *NTRK3*, *SRF*, *VDR*, and *KDR*. These genes were potentially involved in coat color (*KIT*), immune response (*JAK2* and *SOCS1*), heart development (*NTRK3* and *SRF*), muscle growth and development (*VDR*), and fat deposition (*KDR*).

## 4 Discussion

### 4.1 Genetic diversity and population structure of KLPs

Exploring the genetic diversity and population structures of indigenous pig breeds can contribute to their scientific conservation and sustainable development. KLP is a valuable pig resource in southwest China, but its genetic diversity and population structure are still unclear. In this study, a comprehensive analysis was performed by resequencing KLPs and comparing them with the genomic data of DUPs, LRPs, and YRPs. The results showed that KLPs had the largest H_E_, H_O_, P_N_, MAF, and π values, indicating the relatively higher genetic diversity than the other three commercial pig breeds. It is consistent with previous findings from comparative studies between some Chinese indigenous pig breeds and commercial pig breeds (3, 23). This observation could potentially be attributed to the stronger artificial selection pressure imposed on commercial pig breeds relative to Chinese indigenous pig breeds. Compared to other Chinese indigenous pig breeds, the H_E_ and H_O_ values of KLPs (0.3189 and 0.3046) were higher than those of Diannan small-ear pigs (0.2893 and 0.2226) (24), Hechuan black (0.2751 and 0.2958) and Rongchang pigs (0.3012 and 0.3044) (25), while lower than those of Tunchang (0.32 and 0.33) and Dingan pigs (0.32 and 0.34) (3), Pudong White, Erhualian, Meishan, and Jinhua pigs (H_E_ ranged from 0.34 to 0.36, and H_O_ ranged from 0.35 to 0.38) (26). These results indicated that KLPs had a relatively intermediate level of genetic diversity among Chinese indigenous pig breeds. Furthermore, KLPs had the lower total number of ROH and shorter length of ROH per individual among the four breeds, which also reflected the higher genetic variation than DUPs, LRPs, and YRPs. Notably, the length of ROH in KLPs was mainly concentrated in 0.5 ~ 1 Mb (76.67%), and only a few ROHs were larger than 4 Mb. It was speculated that there might have been a high proportion of inbreeding behavior in the early generations of KLPs, while the frequency of inbreeding in recent generations was relatively low. Besides, KLPs had the smallest F_ROH_ value among the four populations. Compared with the previous studies in other Chinese indigenous pigs, the F_ROH_ value of KLPs (0.0479) was higher than that of Liangshan pigs (0.026) (27) and Tunchang pigs (0.0304) (28), but lower than that of Licha black pigs (0.11) (29), Anqing six-end-white pigs (0.17) (30), and Wannan black pigs (0.5234) (31). From the F_ROH_, KLPs exhibited a relatively intermediate level of inbreeding in Chinese indigenous pig breeds, suggesting that effective breeding stock selection and mating strategies should be taken to avoid inbreeding and maintain genetic diversity in KLPs.

The population structure of KLPs was revealed by NJ tree, PCA, ADMIXTURE, IBS genetic distance and G matrices, and LD analysis. According to the results of NJ tree and PCA, KLPs and the three commercial pig breeds were divided into four independent populations. Most individuals in KLPs formed a tight cluster, while a minority were relatively scattered. Meanwhile, the IBS genetic distance and G matrices further indicated that most individuals in KLPs had the distant genetic distances and relationships, and all the individuals were clustered in multiple branches. These results suggested that it was necessary to further strengthen the selection of KLPs to improve the genetic uniformity. Furthermore, the results of the ADMIXTURE analysis were similar to those of the NJ tree and PCA. When K = 4, KLPs were effectively distinguished from DUPs, LRPs, and YRPs, and there was a small amount of genetic components from LRPs and YRPs. This phenomenon might be associated with the historical introduction of LRPs and YRPs, which were subsequently used for crossbreeding with KLPs in the 1950s (6). Based on LD analysis, KLPs showed a higher LD decay, suggesting that KLPs were less affected by selection than the other three breeds.

### 4.2 Selection signatures and candidate genes of KLPs

As one of the unique indigenous pig breeds in China, KLPs have many excellent characteristics owing to the local domestication and selection over hundreds of years. Consequently, some selection signatures likely remain in the genomes of KLPs as a result of domestication. Based on the three comparisons of KLPs with DUPs, LRPs, and YRPs, a total of 688 selected regions were identified, and most of the regions were mainly distributed in chr 8 and 1, which was consistent with the previous study in Anhui local pig breeds (5). Within these selected regions, 723 published QTLs were identified, of which 630 QTLs (87.14%) were associated with carcass traits, fat deposition, and meat quality traits, such as lean cut percentage, number of ribs, longissimus muscle area and depth, subcutaneous fat thickness, meat color, and IMF content, etc. This suggested a strong selection for carcass and meat quality traits during the domestication and breeding of KLPs. It is well known that KLPs exhibit superior meat quality traits (e.g., higher IMF content and water-holding capacity) and adaptability but relatively inferior growth and carcass performance (e.g., lower growth rate, dressing percentage, and lean meat percentage) compared to commercial pig breeds. The overlap of QTLs within the selected regions may provide an explanation for the genetic differences observed between KLPs and the commercial pig breeds.

Within the identified selection regions, 192 candidate genes were annotated. Functional enrichment analyses demonstrated that seven of these candidate genes were consistently present in the top 10 GO terms and KEGG pathways, which might be involved in coat color (*KIT*), immune response (*JAK2* and *SOCS1*), heart development (*NTRK3* and *SRF*), muscle growth and development (*VDR*), and fat deposition (*KDR*).

KIT, also known as C-Kit, is a tyrosine kinase receptor that plays a critical role in melanocyte physiology by influencing melanogenesis, proliferation, migration, and survival of the pigment-producing cells (32). Previous study demonstrated that the deletion of exon 17 of *KIT* attenuated intracellular MAPK and PI3K signaling, impaired migration of embryonic melanoblasts, reduced the number of mature melanocytes, and resulted in a piebald coat color in C57/B6 mice (33). A recent research also showed that *KIT* regulates the melanocyte development and coat color in cat, and that deletion of exon 17 of *KIT* could cause impaired melanoblast proliferation and differentiation (34). In pigs, mutations in *KIT* gene have been shown to affect coat color and color distribution (35), and the selection signatures were also identified in the Chinese Rongchang (36), Taihu (37), and Lulai pigs (38). Coat color is one of the most important characteristics of a breed and used as an exploitable genetic marker. It is known that KLPs predominantly exhibit solid black coat color, with occasional occurrences of six-white (white markings on the head, tail tip, and four hooves) and blond coats (6). The selection of *KIT* gene may provide an explanation for the diversity of coat color phenotypes in KLPs during the domestication.

*JAK2* and *SOCS1* were found to be associated with immune responses. JAK2 is a member of the Janus kinase family, which plays a role in a wide variety of cytokine signaling pathways (39). Research has shown that *JAK2* regulated the development and maturation of dendritic cells, and the secretion of inflammatory cytokines (40). Furthermore, *JAK2* has a crucial function in mammalian immune cell signaling and is associated with immune resistance and escape (41). It was reported that *JAK2* gene was associated with bovine mastitis resistance (42). SOCS1 is a member of the SOCS family that regulates diverse processes, including immune modulation and cell cycle regulation (43). It plays a role in a classic negative feedback loop by inhibiting signaling in response to interferon, interleukin-12, and interleukin-2 family cytokines (44). Studies have shown that *SOCS1* may be a putative candidate gene associated with porcine reproductive and respiratory syndrome virus (PRRSV), and that it could be co-opted to evade the host immune response and facilitate viral replication (45, 46). Unfortunately, there is still a lack of direct and strong evidence for the association between genes *JAK2* and *SOCS1* and disease resistance in pigs. As we know, KLPs have a stronger adaptability and stress resistance than commercial pig breeds. It is valuable to explore whether genes *JAK2* and *SOCS1* are associated with the strong adaptability of KLPs by regulating relative immune processes.

*NTRK3* and *SRF* genes were identified to be related to heart development. NTRK3, also referred to as TRKC, is a neurotrophic tyrosine receptor kinase involved in the nervous system and heart development. *NTRK3* gene encodes the high-affinity receptor neurotrophin-3 (NT-3), which is essential for normal development of the atria, ventricles, and cardiac outflow tracts in mammals (47). An earlier study showed that the TRKC-deficient mice had severe cardiac defects, such as atrial and ventricular septal defects, and valvular defects including pulmonic stenosis (48). It was reported that *TRKC* was expressed by cardiac myocytes and might be responsible for ventricular trabeculation in the first week of chicken development (49). Study has suggested that *NTRK3* played an important role in congenital heart defects, and mutations in *NTRK3* may increase the risk of ventricular septal defect (50). SRF is a critical transcription factor required for the development of cardiomyocytes and plays a central role in heart development and function by regulating genes for cardiac contractile and regulatory proteins (51, 52). Moreover, it acts as a homeostatic regulator between cardiomyocytes and fibroblasts in heart, and dysregulation of SRF is deleterious for this balance (53, 54). Precise regulation of *SRF* expression is critical for mesoderm and cardiac crescent formation in the embryo, and altered SRF levels lead to cardiomyopathies (55). However, no studies have addressed the impact of the two genes on pig heart development. For hundreds of years, KLPs have been raised and domesticated in the high-altitude mountainous regions of Guizhou Province, China. *NTRK3* and *SRF* genes related to heart development was under selection in KLPs, which provided indirect evidence for their better adaptation to the high-altitude harsh environments.

Vitamin D receptor (VDR) plays a crucial role in calcium homeostasis, growth, and differentiation of multiple cell types (56). During skeletal muscle development, VDR plays a physiological role by ensuring the precisely timed downregulation of myogenic transcriptional regulators (57). Study has shown that the overexpression of *VDR* in skeletal muscle resulted in robust myofiber hypertrophy, alongside concurrent gains in protein content synonymous with muscle growth, with increased protein synthesis across muscle protein subfractions (58). It was reported that VDR played a fundamental role in the regulation of myogenesis and muscle mass, whereby it acted to maintain muscle mitochondrial function and limit autophagy (59). Additionally, study in transgenic mice has shown that overexpression of *VDR* in adipocytes resulted in significant increases in body weight gain, fat accumulation and serum lipid levels (60). Research has shown that *VDR* played an important role in adipogenesis in Iberian pigs (61). These results indicate that *VDR* plays an important role in muscle growth and fat deposition. KLPs exhibit relatively slow growth rate, low lean meat percentage (< 42%), and thick carcass backfat (> 45 mm) (6, 8), which may be related to *VDR* gene under selection during the domestication and breeding.

*KDR*, also called *VEGFR2*, encodes a member of the VEGF family that regulates endothelial uptake of fatty acids by controlling the transcription of vascular fatty acid transport proteins (62). As the primary receptor for VEGFA, VEGFR2 activates multiple downstream signaling pathways to mediate angiogenesis (63). Given the reciprocal regulation between adipogenesis and angiogenesis, inhibition of VEGF-VEGFR2 signaling can suppress adipose tissue formation *in vivo* (64). *KDR* gene was reported to be highly expressed in the prothorax and neck adipose tissue of Yanbian yellow cattle (65). Earlier research showed that the mRNA level of *KDR* was significantly correlated with IMF content in *longissimus dorsi* muscle, and the ACA haplotype of genetic variants in the *KDR* transcriptional regulatory region was associated with the higher IMF content in Erhualian pigs (66). Transcriptomic analysis also revealed that *KDR* was a potential candidate gene associated with IMF content in Anqing Six-end-white pigs (67). We speculate that *KDR* gene may be associated with the high IMF content and carcass fat percentage in KLPs, but its exact effect requires further research for confirmation.

## 5 Conclusion

This study revealed that KLPs exhibited higher genetic diversity, a distinct population structure, and significant genetic differentiation among individuals. A total of 688 selected regions were identified, encompassing 723 published QTLs, with 192 candidate genes annotated. Seven genes under selection were found to be involved in coat color (*KIT*), immune response (*JAK2* and *SOCS1*), heart development (*NTRK3* and *SRF*), muscle growth and development (*VDR*), and fat deposition (*KDR*). These findings enable a better understanding of the genomic characteristics and provide valuable references for the conservation, breeding, and utilization of KLPs.

## Data Availability

The datasets generated for this study are publicly available in the China National Center for Bioinformation repository. The link is: https://ngdc.cncb.ac.cn with accession number CRA031687.

## Footnotes

^1^ https://www.ncbi.nlm.nih.gov/sra/?term=

^2^ https://github.com/BGI-shenzhen/VCF2Dis

^3^ http://www.atgc-montpellier.fr/fastme/

^4^ http://genome.ucsc.edu/cgi-bin/hgTables
