# Supplementary material for: Whole-genome resequencing reveals the genetic diversity, population structure and selection signatures in Chinese indigenous Kele pigs
Source: Front Vet Sci. 2025 Oct 29;12:1655561. doi: 10.3389/fvets.2025.1655561 (PMC12605136; doi:10.3389/fvets.2025.1655561)
Supplement: Supplementary file 1 [file Data_Sheet_1.ZIP › Supplementary Material/Supplementary_Material.docx]

Supplementary Material

## Supplementary Figures


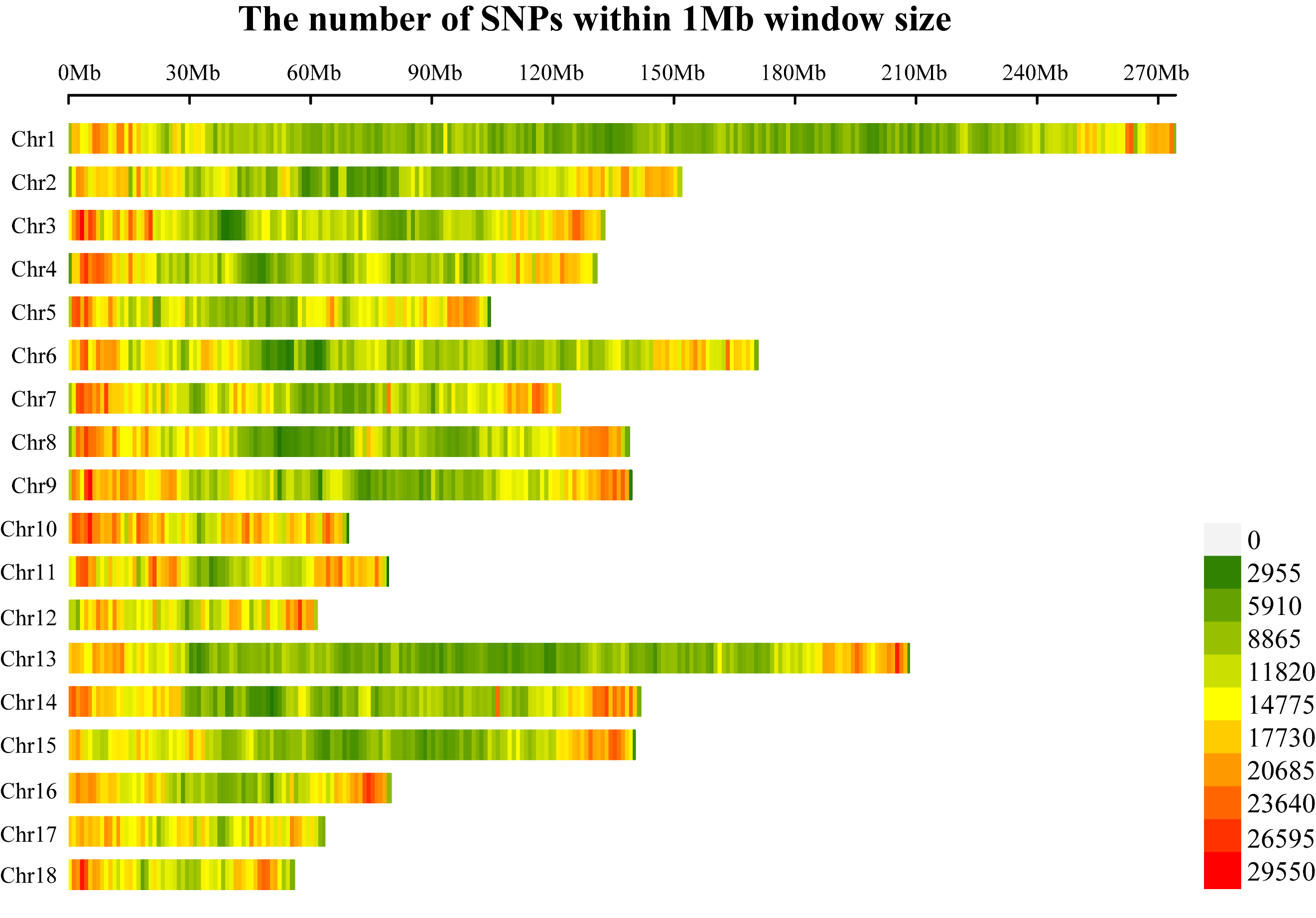


**Supplementary Figure 1.** The plot of the density distribution of SNPs across the chromosomes. The horizontal axis shows the chromosome length (Mb), and different colors represent different density levels.


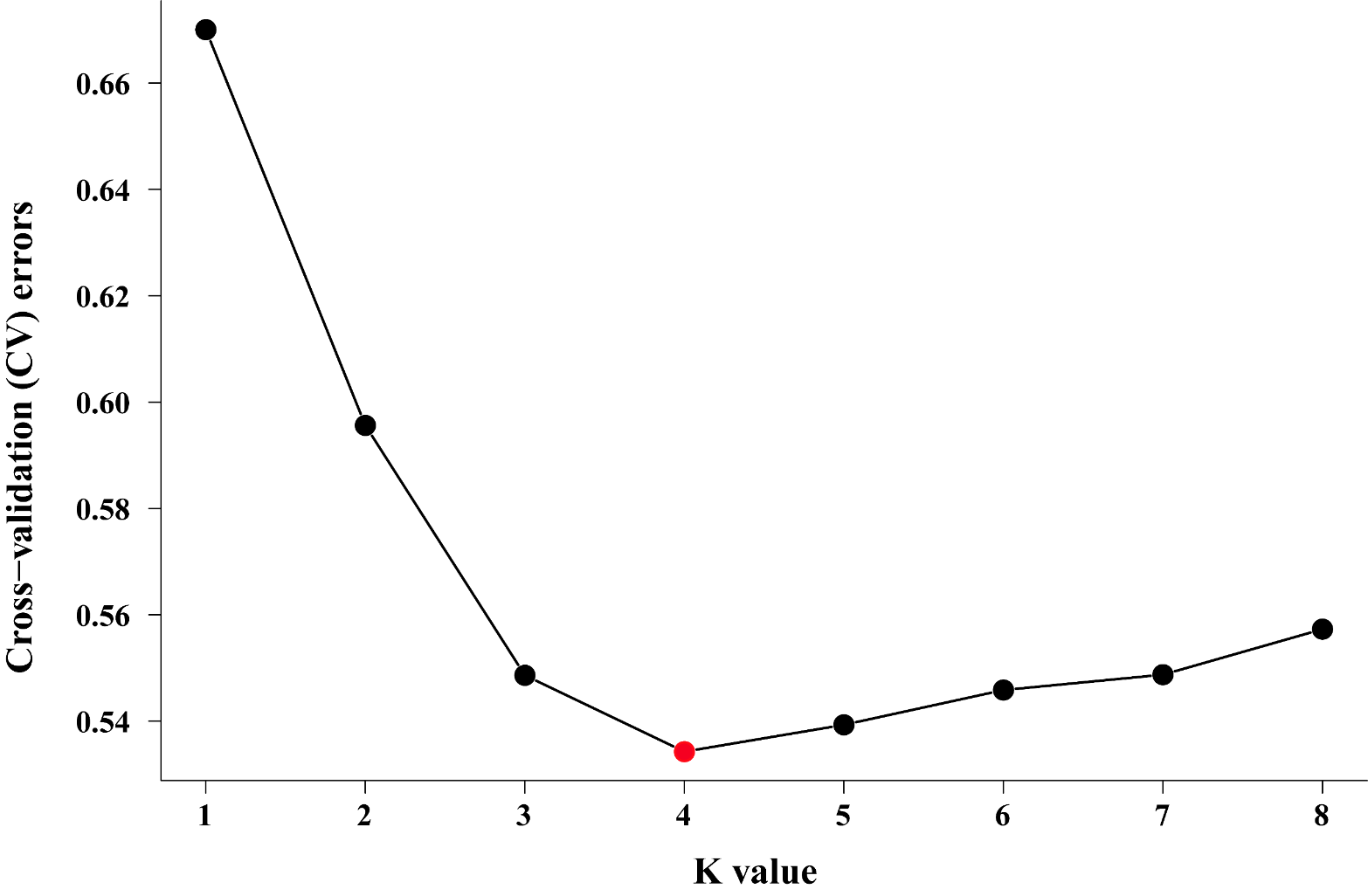


**Supplementary Figure 2.** The plot of cross-validation errors in different K values.
